# Trade Hostility to Hospital Research

**Published:** 1914-06-13

**Authors:** 


### TRADE HOSTILITY TO HOSPITAL RESEARCH.

### The Ideal Antiseptic for Obstetric Work.

THE prevention of puerperal sepsis is a problem which has engaged the attention of a very large number of eminent obstetricians; there is perhaps no topic more frequently discussed in presidential addresses before learned societies connected with this department of practice. In the last few years less attention has been paid in these discussions than was customary a decade ago to the choice of antiseptics for obstetric work. This tendency is justified in that the choice of an antiseptic is a comparatively small part of the whole problem, other aspects of which are of greater importance. Still, it is a part which does not deserve to be completely neglected; and a helpful discussion of it will be found in an article in *American Medicine.**

One sound reason for the disinclination of clinicians and bacteriologists to say very much about the relative merits of various antiseptics may be found in the fact that many of the most popular are patented or trade-marked articles, to comment freely upon which may involve litigation with the owners. It is, admittedly, quite wrong that in a matter so closely affecting the welfare of humanity and the State a scientific worker should have to pick and choose his words or to hold back any part of his observations; but that the danger is not imaginary an incident which took place a few years ago in London very plainly proves. A young and energetic bacteriologist decided to institute comparative tests of the germicidal powers of some four or five antiseptics which were in use at the hospital to which he was attached. He found that one of them, a proprietary article, exhibited this power to a very inferior degree, as far as his researches and tests could estimate it. Full of enthusiasm for the patients and their welfare, he published his researches in the medical school periodical, and advocated that this particular antiseptic should be no longer used in the hospital. Thereupon the manufacturers wrote letters threatening proceedings for damages, and as the editor was not in a financial position to sustain an action he had perforce to publish some sort of disclaimer and apology to satisfy the aggrieved firm. As it happens, that particular antiseptic has since then declined greatly in favour, and is now very seldom heard of; so probably the bacteriologist was perfectly right.

At the present time there are several coal-tar derivatives, mainly proprietary, which are most employed in obstetric practice in this country; though perchloride of mercury, biniodide of mercury, and carbolic acid have also their adherents.

Formalin and its allies, and also the mercurial antiseptics, are taken exception to on the ground that pus, mucus, secretions generally, and soap render them " inert, inefficient, and useless." These antiseptics, it is stated, have no effect upon the vaginal flora, and merely irritate the mucous membrane. Moreover, the mercury salts are highly poisonous, and may be absorbed from raw surfaces in sufficient quantity to cause serious symptoms. There is nothing new, of course, in this observation; but it serves to illustrate the fact that the ideal antiseptic must be non-irritant, non-poisonous, and efficient as a germicide in the presence of organic matter. Chlor-meta-cresol has been favourably reported upon by Burckhardt and Kolb, but there are several preparations which are now said to surpass this product for obstetric practice.

There the article we have quoted leaves the matter. It is to be hoped that the plea for research will be answered; nor is there any reason why the same plea should not be addressed to British investigators. It is discreditable that there should be so much doubt and diversity of practice in the medical profession over the choice of an antiseptic; and the matter is complicated, as has been hinted, by the hostility of manufacturers, all jealous of the sale of their own proprietary articles.

* March 1914, p. 148.

